# There is no association between the presence of anti-thyroid antibodies and increased reproductive loss in pregnant women after ART: a systematic review and meta-analysis

**DOI:** 10.5935/1518-0557.20170057

**Published:** 2017

**Authors:** Paz Leiva, Juan Enrique Schwarze, Pamela Vasquez, Carolina Ortega, Sonia Villa, Javier Crosby, José Balmaceda, Ricardo Pommer

**Affiliations:** 1 Obstetrics and Gynecology Department at Universidad de Santiago, Chile; 2 Reproductive Medicine Unit at Clinica Monteblanco; 3 Epidemiology Department, Universidad de los Andes, Chile; 4 Reproductive Medicine Unit at Clinica Las Condes

**Keywords:** ART, anitbodies, thyroid

## Abstract

Women submitted to ART treatments represent a select subgroup of individuals. Several studies have described the relationship between TAI and pregnancy outcomes as a result of ART, with contradictory results. The purpose of this systematic review was to determine the association between TAI and the risk of miscarriage in pregnancies resulting from ART. MEDLINE via PubMed, LILACS and Embase were searched for studies published in peer-reviewed journals from 1999 to 2017. The studies were summarized using the fixed effects model and the Peto's method to calculate RR in order to flesh out the association between TAI and spontaneous abortion. Only four papers were included in this systematic review and meta-analysis. Thirty-one miscarriages were observed in 210 clinical pregnancies of women with antithyroid antibodies; and 158 miscarriages were seen in 1,371 pregnancies without antithyroid antibodies. The meta-analysis failed to find an association between TAI and higher risk of reproductive loss, RR=0.94 95% confidence interval: 0.71-1.24; *p*=0.879. In conclusion, the presence of antithyroid antibodies was not associated with increased reproductive loss in patients submitted to ART treatments. It is our opinion that the presence of antithyroid antibodies should be considered as a secondary biomarker of autoimmune disease, rather than an actual cause of miscarriage in patients undergoing ART. Due to the small amount of evidence on the matter, the determination of TAI before the initiation of ART should be limited to research contexts.

## INTRODUCTION

The percentage of women in the general population with thyroid autoimmunity (TAI), whether by thyroglobulin autoantibodies (anti-Tg) or anti-thyroid peroxidase antibodies (TPOAb), may be as high as 20% (Davies, 2016; Łukaszuk *et al.*, 2015). In 1930, Stagnaro-Green *et al.* (1990) described the association between TAI and risk of spontaneous abortion. Since then, increased risk of fetal loss, perinatal mortality, and large for gestational age (LGA) newborns have been reported for euthyroid women with elevated concentrations of TPOAb (Bussen & Steck, 1995; Prummel & Wiersinga, 2004; Männistö *et al*. 2009). Other studies suggested that the presence of TAI in euthyroid women was associated with a 2-3 fold higher risk of miscarriage (Chen & Hu, 2011; Thangaratinam *et al*., 2011). The causality and pathophysiology of this association, or the adequate course of treatment, have not been completely elucidated.

Women submitted to assisted reproductive technology (ART) treatments represent a select subgroup of individuals. Several studies have described the relationship between TAI and pregnancy outcomes as a result of ART, with contradictory results (Abalovich *et al.*, 2007; Stagnaro-Green & Glinoer, 2004). The purpose of this systematic review was to determine the association between TAI and the risk of miscarriage in pregnancies resulting from ART.

## MATERIALS AND METHODS

### Electronic search

MEDLINE via PubMed, LILACS and Embase were searched for papers written in English and Spanish published in peer-reviewed journals from 1999 to 2017. Search terms "antithyroid antibodies" "assisted reproductive techniques", and the combined MeSH terms "reproductive techniques, assisted OR fertilization *in vitro* OR sperm injections, intracytoplasmic" AND "thyroid gland" AND "autoantibodies" were used. Additional studies were found in the references of the retrieved papers.

### Study selection

Studies looking into ART in women with ages ranging from 22 to 45 years were eligible for inclusion in the review. Secondary studies, studies without comparable groups, and studies performing Preimplantation Genetic Diagnosis were excluded.

**Figure 1:**
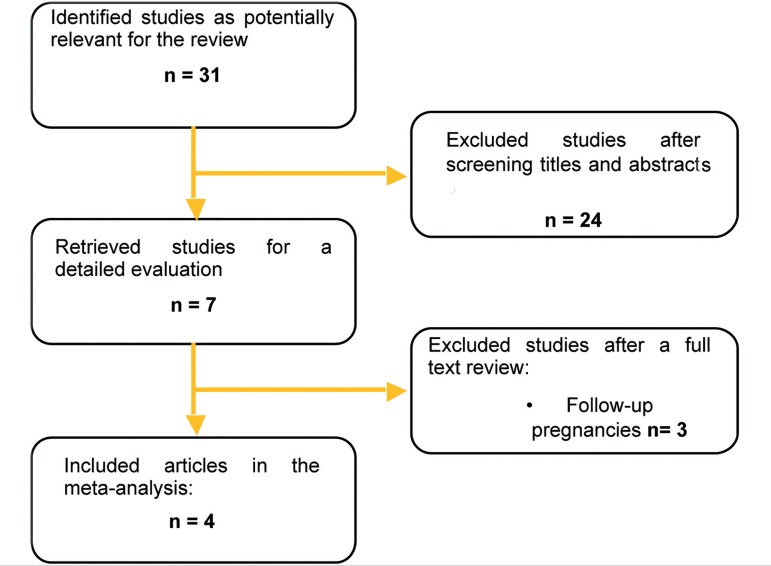
Systematic review flowchart.

The papers were selected based on their titles and abstracts according to the inclusion criteria (Figure 1).

### Selected outcomes

Papers comparing the pregnancy outcomes of individuals with and without TAI offered ART treatments were included, whereas studies not reporting pregnancy outcomes were not included (e.g. birth or miscarriage).

### Data extraction

Two independent authors reviewed the titles and abstracts (PL and JES), and when applicable the full text was retrieved for further analysis. The independent authors assessed the papers for compliance with the inclusion criteria. Disagreements were resolved with the aid of a third author (CO). Data was extracted by one of the authors (PL) in a specially designed form that included references, study type, methods, results, and conclusions.

### Methods of synthesis

**Figure 2:**
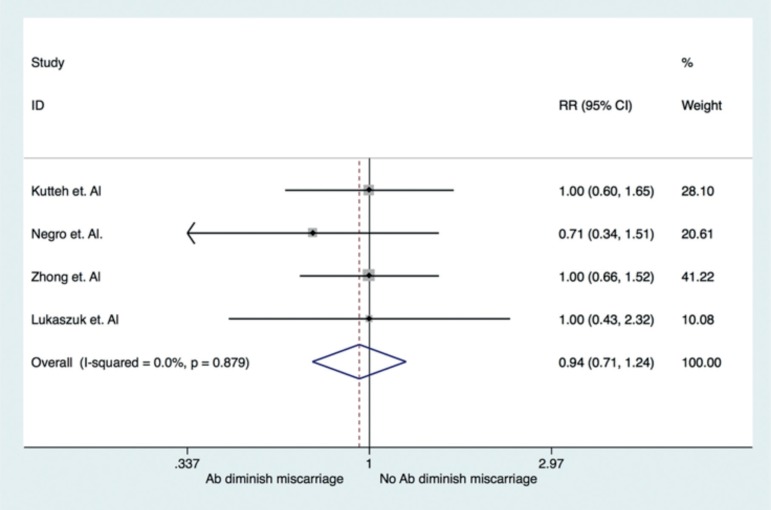
Forest plot: RR of miscarriage in TAI (+) patients undergoing ART.

The studies were summarized using the fixed effects model and Peto´s method to calculate relative risk (RR) and 95% confidence intervals to further elicit the association between TAI and spontaneous abortion in women offered ART. Statistical analysis was performed on STATA 11.0 (STATA Corp, EEUU). The results were displayed in a forest plot (Figure 2).

Heterogeneity between studies was assessed with Higgins' I^2^ and Cochran's Q test. Heterogeneity was considered to be significant when *p*<0.01 and I^2^>30%.

## RESULTS

The initial search retrieved 31 studies. No duplicates were found. After screening for titles and abstracts, 24 studies were excluded for not meeting the inclusion criteria. Seven remained for full text revisions, but three included ongoing pregnancies and were therefore excluded. Four papers met the inclusion criteria and were included in the systematic review and meta-analysis (Figure 2).

The four studies included were published between 1999 and 2015 and covered a total of 2,664 women offered ART. The main findings are summarized in Table 1. Two of the studies were carried out in Europe, one in Asia, and one in the United States. All were retrospective cohort studies.

**Table 1:** Summary of included studies.

Author, year. Country	Methods	TAI (+)	TAI (-)	Conclusions
**Lukaszuk *et al.*, 2015. China**	Retrospective Cohort study of euthyroid women, age 34-35 years, submitted to IVF/ICSI between April 2010 and April 2012. Population was divided in 2 groups: patients with TPOAb+ (n=114) and TPOAb- (n=495) Measurement of TPOAb by ECLIA. Reference value: 0-34 IU/ml	**Clinical Pregnancy:** n=50 **Miscarriage: ** n=3	**Clinical Pregnancy: ** n=235 **Miscarriage: ** n=29	IVF patients undergoing ICSI with TPOAb+ vs TPOAb- did not present statistically significant differences in fertilization, implantation, pregnancy, and live newborn rates. The presence of TPOAb did not increase the risk of miscarriage (6% vs. 12.4%, *p*=0.29)
**Zhong *et al.*, 2012. Poland**	Retrospective cohort study of patients (mean age=32 years) submitted to IVF/ICSI between August 2009 and August 2010. Population was divided in two groups: patients with TAI [TPOAb+ and/or anti-Tg+] (n=90) and without TAI (n=676) Measurement of TAI by CMIA: -TPOAb+ ≥561UI/ml -anti-Tg+] ≥4.11UI/ml	**Clinical Pregnancy:** n=52 **Miscarriage**: n=14	**Clinical Pregnancy: ** n=458 **Miscarriage**: n=54	Fertilization implantation and pregnancy rates after IVF-ET were significantly lower (64.3% vs. 74.6%, *p*<0.001; 17.8% vs. 27.1%, *p*<0.001; and 33.3% vs. 46.7%, *p*=0.002, respectively), whereas miscarriage rates were significantly higher (26.9% vs. 11.8%) in patients with TAI versus controls (without TAI).
**Negro *et al*., 2007 Italy**	Retrospective Cohort study of euthyroid women aged 20-35 years, carried out between January 2000 and January 2005. A total of 416 patients were selected; 42 had TPOAb+ and 374 TPOAb-. Measurement method of TPOAb by RIA: -TPOAb+ ≥100 Ku/l	**Clinical Pregnancy: ** n=21 **Miscarriage**: n=5	**Clinical Pregnancy: ** n=234 **Miscarriage**: n=27	In euthyroid patients, pregnancy and delivery rates were not affected by the presence of TPOAb. In patients with TPOAb+, the subgroup of patients that did not achieve pregnancy had miscarriages had higher TSH levels, but within the normal range (2.8mUI/ml) vs. patients that delivered (TSH 1.06 mUI/ml, *p*=0.032)
**Kutteh *et al.*, 1999 USA**	Retrospective cohort study of women aged 35±4 years offered IVF in 3 centers in the USA between April 1996 and April 1997. From a total of 873 patients with ART, 143 women had TAI [TPOAb+ and/or anti-Tg+]. Results were compared to a control group of 200 non-pregnant women of childbearing age with no record of reproductive problems Measurement of AIT with ELISA: -TPOAb+ ≥65 UI/ml. -anti-Tg+ ≥120 UI/ml.	**Clinical Pregnancy** n=87 **Miscarriage**: n=9	**Clinical Pregnancy**: n=444 **Miscarriage**: n=48	The presence of TAI was similar between patients offered ART and controls (16.4% vs. 14.5 %, OR: 1.16) No statistically significant differences were found in delivery (54.5% vs. 54.2%, *p*=1.00), biochemical miscarriage (3.5% vs. 4.7%, *p*=0.66), clinical miscarriage (6.3% vs. 6.6%, *p*=1.00), and pregnancy failure (35.7% vs. 34.5%, *p*=0.85) rates between patients with and without TAI.

The groups were comparable for age in the selected papers. Only two studies mentioned the nutritional status of the patients with a mean body mass index of 22±4Kg/m^2^ (Łukaszuk *et al*., 2015; Zhong *et al*., 2012); two included only euthyroid women and two did not consider that condition, although they excluded individuals with other autoimmune diseases.

The methods used to determine the presence of TAI differed, as did their titrations: electrochemiluminescence immunoassay (Łukaszuk *et al.*, 2015), chemiluminescent microparticle immunoassay (Zhong *et al*., 2012), radioimmunoassay (Negro *et al*., 2007), and enzyme-linked immunosorbent assay (Kutteh *et al*., 1999) (Table 1). In two studies the anti-Tg and anti-thyroid peroxidase antibodies levels were measured (Łukaszuk *et al*., 2015; Negro *et al.,* 2007), while the other two only the level of anti-thyroid peroxidase antibodies was measured (Kutteh *et al.*, 1999; Zhong *et al.,* 2012).

Thirty-one miscarriages were observed in 210 clinical pregnancies of women with antithyroid antibodies; and 158 miscarriages were seen in 1,371 pregnancies without antithyroid antibodies. The meta-analysis failed to find an association between TAI and higher risk of reproductive loss, RR=0.94 95% confidence interval: 0.71-1.24; *p*=0.879 (Figure 2).

The heterogeneity between studies was not significant (*p*=0.879, I^2^=0.00%).

## DISCUSSION

The purpose of this review was to determine whether pregnant individuals with TAI offered ART were at higher risk of having a miscarriage. After summarizing four studies including a total of 1,581 pregnancies after ART, no association was found between TAI and miscarriage. Most of the studies included in this meta-analysis showed no statistically significant differences in fertility rate, number of embryos available, implantation rate or clinical pregnancy rates between the groups. These findings were consistent with the results reported by Karacan *et al*. (2013), in which implantation rates, spontaneous abortion rates, and pregnancy rates did not differ significantly between the groups with TAI and without TAI.

The strength of this study resided in the large number of analyzed patients, a total of 2,664 individuals given ART treatments. A weakness of the study was the fact that levels of thyroglobulin autoantibodies (anti-Tg) were not measured in every included study, which have been reported to be around 5% in patients suffering from infertility (Unuane *et al.,* 2013). Another limitation was that only two studies included euthyroid patients; the other two did not consider this trait in their inclusion criteria, but had presence of other autoimmune diseases as a criterion for exclusion. Levothyroxine was not prescribed to every patient included. Since TSH levels and prescription of levothyroxine seem to be relevant for IVF outcomes, Poppe *et al*. (2004) looked into the thyroid function of females submitted to IVF procedures and the possible associations with reproductive outcomes, and reported a modified pattern in thyroid function during the first period of pregnancy after comparing the groups with and without TAI.

Most of the patients did not receive levothyroxine during pregnancy. The meta-analysis performed by Velkeniers *et al*. (2013) and the study by Negro *et al*. (2005) found that thyroxine supplementation for women with subclinical hypothyroidism and/or thyroid autoimmunity might improve clinical pregnancy outcomes in patients offered ART, therefore such supplementation did not interfere with the outcomes described in our study.

The discrepancies between the results found in this study and in the papers written by Zhong *et al*. (2012) and Glinoer *et al*. (1991) may be explained by the mechanism of action of antithyroid antibodies; although this mechanism has not been completely described, it has been speculated that the anti-thyroid peroxidase antibodies can bind to the egg surface and/or embryo and interfere with fertilization and embryo development (Monteleone *et al.,* 2011; Zhong *et al.,* 2012). Our results, although contradictory at a first glance, seem to confirm this hypothesis: While TPOAb bound to the egg surface might prevent sperm cells from entering the egg during natural fertilization or IVF, it should not affect fertilization through intracytoplasmic sperm injection (ICSI), the method used in all patients in the included studies.

In conclusion, the presence of antithyroid antibodies was not associated with increased reproductive loss in patients submitted to ART treatments. It is our opinion that the presence of antithyroid antibodies should be considered as a secondary biomarker of autoimmune disease, rather than an actual cause of miscarriage in patients undergoing ART. Due to the small amount of evidence on the matter, the determination of TAI before the initiation of ART should be limited to research contexts.
